# *In Vitro* Cytotoxic Activity of *Origanum vulgare* L. on HCT-116 and MDA-MB-231 Cell Lines

**DOI:** 10.3390/plants2030371

**Published:** 2013-06-25

**Authors:** Filip Grbović, Milan S. Stanković, Milena Ćurčić, Nataša Đorđević, Dragana Šeklić, Marina Topuzović, Snežana Marković

**Affiliations:** 1 Department of Biology and Ecology, Faculty of Science, University of Kragujevac, Str. Radoja Domanovića No. 12, 34000 Kragujevac, Republic of Serbia; 2 Department of Biomedical Sciences, State University of Novi Pazar, Vuka Karadzica, 36300 Novi Pazar, Republic of Serbia

**Keywords:** antiproliferativeactivity, HCT-116, MDA-MB-231, *Origanum vulgare* L.

## Abstract

In the present investigation, we examined the cytotoxic effect of methanolic extract from *Origanum vulgare* on HCT-116 and MDA-MB-231 cell line *in vitro*. In order to determine the cytotoxic effects we used an MTT viability assay. The results showed that cell growth is significantly lower in extract treated cells compared to untreated control. The effect of inhibition of cell growth was higher in the treatment of HCT-116 cell line than in MDA-MB-231. Based on the results it is determined that *O. vulgare* is a significant source of biologically active substances that have cytotoxic and antiproliferative activity *in vitro*.

## 1. Introduction

**Figure 1:**
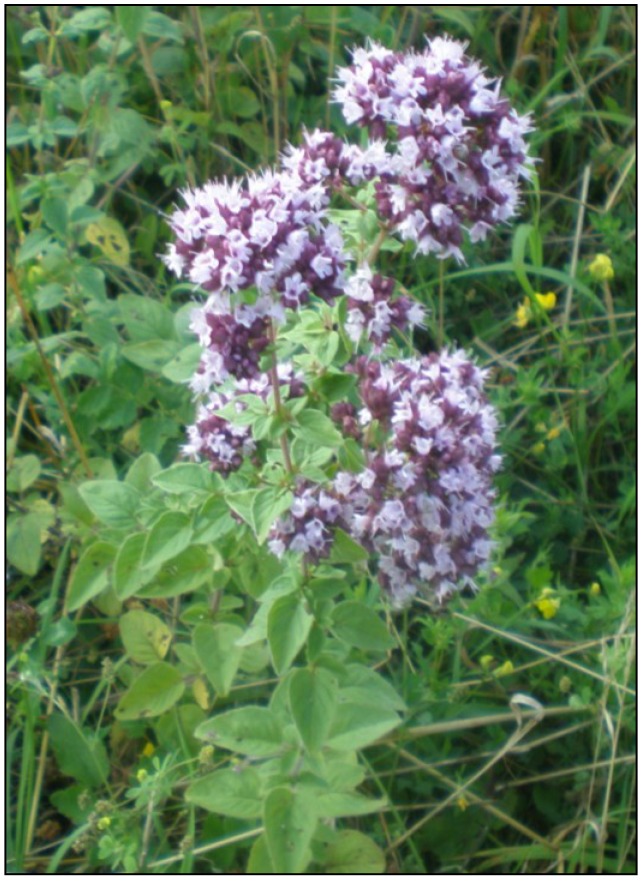
*Origanum vulgare* L. in flowering stage.

*Origanum vulgare* L. is a perennial herbaceous plant belonging to the family Lamiaceae (Figure 1). The common name of this species is oregano. The scientific name of this plant is derived from two Greek terms—oros (mountain) and ganos—(joy, beauty, decoration). It is 20–80 cm tall, with opposite leaves 1–4 cm long. Its short-stem flowers are usually purple, and 4–7 mm long. It occurs on dry slopes, poor meadows, in lighter deciduous forests, thickets, and openings in forests up to 2,000 m above sea level. It is widespread in Europe and Asia [1].

*O. vulgare* is a well-known culinary herb whose leaves can enhance the flavor of food. The species is also used in traditional and modern medicine and in pharmaceutical industry. In a number of studies, large amounts of active secondary metabolites as well as a variety of biological activities of this plant were evaluated [2,3].

Throughout history, man has striven to find ingredients that may help treat diseases and maintain health in the nature as an inexhaustible source of new biologically active compounds. Most medicinal substances originate from different wild plants. Ingredients of many plants have antiseptic, antibiotic, and antioxidant properties [4]. There is also a vast array of plant species used in cancer prevention and treatment due to the anticancer compounds they contain [5].

The aim of this study was to examine cytotoxic activity of methanolic extracts obtained from *O. vulgare*. It seems that very little data on cytototoxicity and antitumour activities of this plant exist in literature. Colon cancer (HCT-116) and breast cancer (MDA-MB-231) have been selected for the research as they are leading causes of cancer death.

## 2. Results and Discussion

Cancer-related research is conducted worldwide every day, since cancer is a leading cause of death. These studies often involve the investigation of the effects of biologically active substances on cancer cells, and they frequently originate from plants [6]. There is a great need to examine reliable and inexhaustible sources of natural substances. In addition, it is important to understand the mechanisms of anticancer agents for future application in cancer therapy [7].

This experiment investigated the cytotoxic activity of the methanolic extract of *O. vulgare* (in a concentration of 1–500 µg/mL) in two cell lines (HCT-116 and MDA-MB-231), using MTT assay. A dose-dependent MTT reduction (or color change from yellow to purple) was observed in both extract-treated cell lines. 

**Figure 2:**
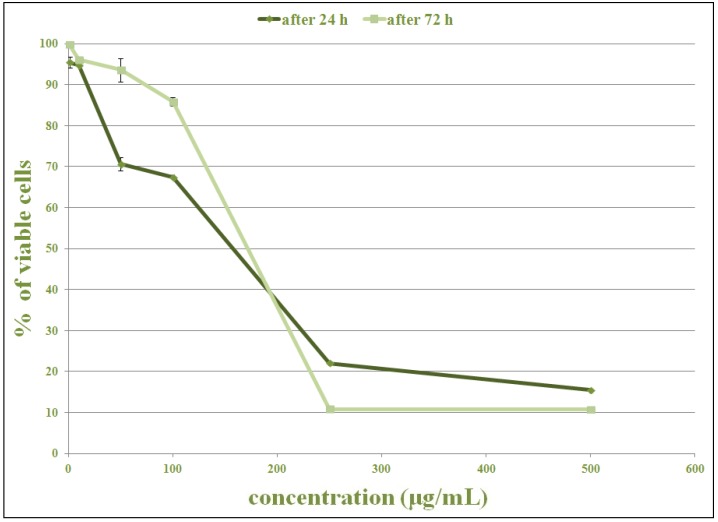
The cytotoxic effect of methanolic extract of *O. vulgare* on HCT-116 cells, 24 and 72 h after exposure. The effect was measured by MTT cell viability assay. The data are mean ± SD of three independent experiments.

The shape of the curve shows significant inhibition of cell proliferation in HCT-116 cell line (Figure 2) in a dose-dependent manner after 24 and 72 h of treatment. The cell proliferation was significantly lower (*p* < 0.05) when compared to untreated control cells. After 24 h of treatment, higher concentrations (250 and 500 µg/mL) killed more than 50% of cells. After 72 h of treatment cytotoxic effect of those concentrations was higher, but lower concentrations had weaker cytotoxic effects than after 24 h. Maximum inhibition of proliferation was achieved after 72 h at the highest concentration (500 µg/mL).

**Figure 3:**
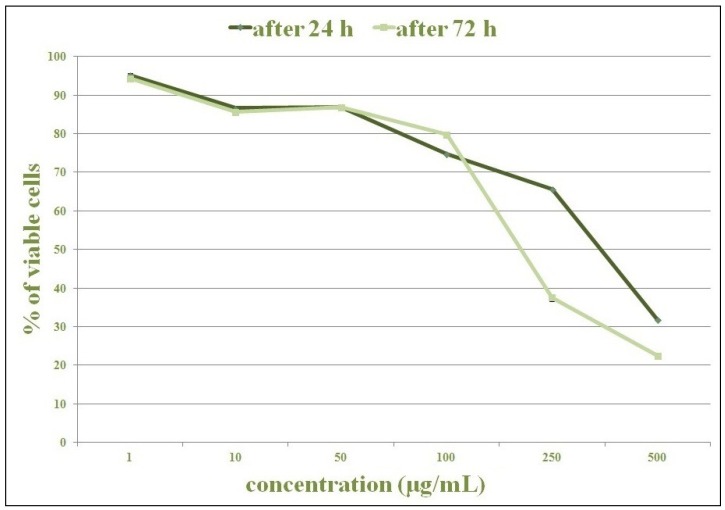
The cytotoxic effect of methanolic extract of *O. vulgare* on MDA-MB-231 cells, 24 and 72 h after exposure. The effect was measured by MTT cell viability assay. The data are mean ± SD of three independent experiments.

Significantly lower cytotoxic activity was also reported in the treatment of MDA-MB-231 cell lines (Figure 3), when compared to untreated control cells. As in HCT-116 cell lines, after 24 h only the highest concentration (500 µg/mL) killed more than 50% of cells. After 72 h the concentration of 250 µg/mL led to the death of more than half of the cells. Maximum inhibition of proliferation was achieved after 72 h at the highest concentration (500 µg/mL).

The results indicated more dramatic effects of cytotoxic activity after the treatment with extract *O. vulgare* on HCT-116 cell lines than on MDA-MB-231 cells. Only 10% of the HCT-116 cells remained viable 72 h after the treatment with extract from *O. vulgare* at a concentration of 250 µg/mL. The same concentration of the plant extract (250 µg/mL) killed 67% of the cells (33% of viable cells) in the treatment of MDA-MB-231 cell lines after 72 h. These results suggest greater effectiveness of the extract from *O. vulgare* on HCT-116 cell line.

Table 1 presents *in vitro* cytotoxic activity. The effects of extract were expressed by IC_50_ values (concentration which inhibit 50% of cell growth), as a parameter for cytotoxity.

**Table 1:** Growth inhibitory effects—IC_50_ values (μg/mL) of methanolic extract of *O. vulgare* on HCT-116 and MDA-MB-231 cell lines after 24 and 72 h exposure.

Cell lines	24 h	72 h
HCT-116	140.77 ± 2.13	109.51 ± 1.28
MDA-MB-231	231.46 ± 1.15	506.31 ± 4.13

The results indicate that *O. vulgare* is considered to be a particularly valuable source of effective anti-proliferative and cytotoxic substances. Previous research of *O. vulgare* in Serbia [8,9] and Greece [10,11] has indicated high concentration of phenolic compounds, responsible for high antioxidative, soybean lipoxygenase activity. In studies of quantitative composition of secondary metabolites from *O. vulgare* ssp. *hirtum*, apigenin, luteolin, chrysoeriol, diosmetin, quercetin, eriodictyol, cosmoside, vicenin-2, caffeic acid, p-menth-3-ene-1,2-diol 1-*O*-beta-glucopyranoside, thymoquinol 2-*O*-beta-glucopyranoside, thymoquinol 5-*O*-beta-glucopyranoside, thymoquinol 2,5-*O*-beta-diglucopyranoside, 12-hydroxyjasmonic acid, 12-hydroxyjasmonic acid 12-*O*-beta-glucopyranoside, lithospermic acid B, rosmarinic acid, 10-epi-lithospermic acid and epi-lithospermic acid B were identifed as polar constituents [12]. 

Many plant extracts and natural products, especially phenolics, with high antioxidant activity have shown cytotoxic effects in different cell lines [13]. 

Consumption of foods that contain phenolics and flavonoids has been shown to reduce the risk of cancer [14]. In addition, several studies have indicated high cytotoxic and anticancer activity of flavonoids [15,16]. Studies have shown that flavonoids may reduce breast cancer cell proliferation [17,18] and human colon carcinoma cell proliferation [19]. Flavonoid anticancer activities include inhibition of cell growth, inhibition of protein kinase activities, induction of apoptosis [20].

Based on these arguments, we can conclude that the high concentration of phenolics and flavonoids is most likely responsible for the significant cytotoxic activity of *O. vulgare* methanol extract. Further research should be carried out to isolate and identify biologically active substances from *O. vulgare*, with an antiproliferative activity, as well as to conduct a detailed examination of the effects of these agents on various cancer cell lines and *in vivo* tests.

## 3. Experimental Section

### 3.1. Chemicals

Dublecco’s Modified Eagle Medium (DMEM) was obtained from GIBCO, Invitrogen, USA. Fetal bovine serum (FBS) and trypsin-EDTA were from PAA (The cell culture company), Austria. Dimethyl sulfoxide (DMSO) and 3-[4,5-dimethylthiazol-2-yl]-2,5-diphenyltetrazolium bromide (MTT) were obtained from SERVA, Germany. All other solvents and chemicals were of analytical grade.

### 3.2. Plant Material

Aboveground plant parts of *Origanum vulgare* L. were collected in Avgust 2010 from the region of Pčinja river gorge in south Serbia. The voucher specimen of *O. vulgare* was confirmed and deposited in Herbarium at the Department of Biology and Ecology, Faculty of Science, University of Kragujevac. The collected plant parts were air-dried in darkness at room temperature (20 °C). 

### 3.3. Preparation of Plant Extracts

The prepared plant material from *O. vulgare* (10 g) was broken in the small pieces 2–6 mm by using a cylindrical crusher and extracted with methanol using Soxhlet apparatus. The extract was filtered through a filter paper (Whatman, No. 1) and evaporated. The residue was stored in a dark glass bottle for further processing.

### 3.4. Cytotoxic Activity

#### 3.4.1. Cell Preparation and Culturing

HCT-116 and MDA-MB-231 cell lines were obtained from American Type Culture Colection. Cells were maintained in DMEM supplemented with 10% Fetal Bovine Serum, with 100 units/mL penicillin and 100 µg/mL streptomycin. Cells were cultured in a humidified atmosphere with 5% CO_2_ at 37 °C. Cells were grown in 75 cm^2^ culture bottles supplied with 15 mL DMEM, and after a few passages cells were seeded in 96-well plate. All studies were done with cells at 70 to 80% confluence. 

#### 3.4.2. Cell Viability Assay (MTT Assay)

HCT-116 and MDA-MB-231 cells were seeded in a 96-well plate (10 000 cells per well). After 24 h of cells incubation, the medium was replaced with 100 μL medium containing various doses of methanolic extracts of *O. vulgare* at different concentrations (1, 10, 50, 100, 250 and 500 μg/mL) for 24 and 72 h. Untreated cells served as the control. 

After 24 and 72 h of treatment the cell viability was determined by MTT assay [17]. The proliferation test is based on the color reaction of mitochondrial dehydrogenase in living cells by MTT. At the end of the treatment period, MTT (final concentration 5 mg/mL PBS) was added to each well, which was then incubated at 37 °C in 5% CO_2_ for 2–4 h. The colored crystals of produced formazan were dissolved in 150 μL DMSO. The absorbance was measured at 570 nm on Microplate Reader. Cell proliferation was calculated as the ratio of absorbance of treated group divided by the absorbance of control group, multiplied by 100 to give a percentage proliferation. 

### 3.5. Statistical Analysis

The data is expressed as means ± standard errors (SE). Biological activity was examined in three individual experiments, performed in triplicate for each dose. Statistical significance was determined using the Student’s *t*-test or the one-way ANOVA test for multiple comparisons. A *p* value < 0.05 was considered as significant. The magnitude of correlation between variables was done using a SPSS (Chicago, IL, USA) statistical software package (SPSS for Windows, ver. 17, 2008). The IC_50_ values were calculated from the dose curves by a computer program (CalcuSyn). 

## 4. Conclusions

Based on these results, we conclude that the methanolic extract of *Origanum vulgare* showed significant antiproliferative activity in both HCT-116 and MDA-MB-231 cell lines. Inhibitory activity appeared to be particularly conspicuous in treated HCT-116 cell lines, whereas at the highest concentration (500 µg/mL), only 10% of the cells remain viable. These data indicate high antiproliferative potential of biologically active substances extracted from *Origanum vulgare*.
